# Transcriptomic changes in *Cucurbita pepo* fruit after cold storage: differential response between two cultivars contrasting in chilling sensitivity

**DOI:** 10.1186/s12864-018-4500-9

**Published:** 2018-02-07

**Authors:** F. Carvajal, R. Rosales, F. Palma, S. Manzano, J. Cañizares, M. Jamilena, D. Garrido

**Affiliations:** 1 Department of Plant Physiology, Facultad de Ciencias, University of Granada, Fuentenueva s/n, 18071 Granada, Spain; 2 Department of Biology and Geology, Agrifood Campus of International Excellence (CeiA3), CIAIMBITAL, University of Almería, La Cañada de San Urbano s/n, 04120 Almería, Spain; 3 Institute for the Conservation and Breeding of Agricultural Biodiversity (COMAV-UPV), Universitat Politécnica de Valencia, Camino de Vera s/n, 46022 Valencia, Spain

**Keywords:** Zucchini fruit, Postharvest physiology, Cold tolerance, Transcriptomic profiling, Stress response

## Abstract

**Background:**

Zucchini fruit is susceptible to chilling injury (CI), but the response to low storage temperature is cultivar dependent. Previous reports about the response of zucchini fruit to chilling storage have been focused on the physiology and biochemistry of this process, with little information about the molecular mechanisms underlying it. In this work, we present a comprehensive analysis of transcriptomic changes that take place after cold storage in zucchini fruit of two commercial cultivars with contrasting response to chilling stress.

**Results:**

RNA-Seq analysis was conducted in exocarp of fruit at harvest and after 14 days of storage at 4 and 20 °C. Differential expressed genes (DEGs) were obtained comparing fruit stored at 4 °C with their control at 20 °C, and then specific and common up and down-regulated DEGs of each cultivar were identified. Functional analysis of these DEGs identified similarities between the response of zucchini fruit to low temperature and other stresses, with an important number of GO terms related to biotic and abiotic stresses overrepresented in both cultivars. This study also revealed several molecular mechanisms that could be related to chilling tolerance, since they were up-regulated in cv. Natura (CI tolerant) or down-regulated in cv. Sinatra (CI sensitive). These mechanisms were mainly those related to carbohydrate and energy metabolism, transcription, signal transduction, and protein transport and degradation. Among DEGs belonging to these pathways, we selected candidate genes that could regulate or promote chilling tolerance in zucchini fruit including the transcription factors MYB76-like, ZAT10-like, DELLA protein GAIP, and AP2/ERF domain-containing protein.

**Conclusions:**

This study provides a broader understanding of the important mechanisms and processes related to coping with low temperature stress in zucchini fruit and allowed the identification of some candidate genes that may be involved in the acquisition of chilling tolerance in this crop. These genes will be the basis of future studies aimed to identify markers involved in cold tolerance and aid in zucchini breeding programs.

**Electronic supplementary material:**

The online version of this article (10.1186/s12864-018-4500-9) contains supplementary material, which is available to authorized users.

## Background

Refrigerated storage is considered to be the most effective method for preserving the quality of fruit and vegetables, allowing long-distance transport and thus a more regulated supply of commodities in the market. However, fruit from tropical and subtropical origins are prone to chilling injury (CI) during storage at low, non-freezing temperatures, as is the case for zucchini squash (*Cucurbita pepo* L.). Fruit from this species are marketed at an immature stage and are susceptible to developing CI symptoms when stored at low temperature, including peel pitting, weight loss, and softening [1, 2]. Previous research has focused on unraveling some of the mechanisms associated with the response of zucchini fruit to cold stress, revealing that the exposure of zucchini fruit to chilling results in a series of ultra-structural, physiological, and biochemical modifications common to other stresses such as an accumulation of hydrogen peroxide (H_2_O_2_) and malondialdehyde (MDA), as well as changes in the levels of endogenous abscisic acid [3, 4], ethylene [5, 6], polyamines [7] and in soluble sugars [8]. Moreover, previous works have demonstrated the existence of genetic variability for CI tolerance among commercial and local cultivars of zucchini squash [6, 9]. The fruit of the most CI-tolerant cultivars produces less chilling-induced ethylene and accumulates lower content of H_2_O_2_ and MDA, two metabolites associated with oxidative stress. Among commercial hybrids, fruit from Natura showed very low CI after 14 days of cold exposure, while fruit from cv. Sinatra presented high CI index and an important loss of fruit quality [9]. Subsequent physiological research revealed many differences between these two cultivars in some metabolic pathways involved in chilling stress and tolerance, confirming that Natura and Sinatra should be considered tolerant and sensitive cultivars to cold stress, respectively. In this sense, Natura fruit has higher soluble sugar content, higher levels of proline, lower content in chilling stress metabolites such as H_2_O_2_ or MDA, and higher gene expression and activity of antioxidant defense enzymes, than Sinatra fruit [7, 10, 11]. In relation to the hormones involved in cold tolerance, it has been reported that the fruit of the most CI-tolerant cultivars shows a reduced induction of ethylene biosynthesis and signaling pathways under cold storage, and that the treatment with 1-MCP prevents chilling damage [6, 11]. Recently we have also detected an increase in the synthesis of abscisic acid (ABA) during the first days of cold storage in the more cold-tolerant cultivar [3]. Hence, there is an increasing amount of information concerning the physiology and biochemistry of chilling in zucchini fruit, however, the molecular mechanisms underlying the response of zucchini fruit to chilling storage is limited to specific molecular pathways, including those of ethylene biosynthesis and signaling [5, 6], enzymatic antioxidant system [12, 13], abscisic acid synthesis and signaling, and polyamine metabolism [3, 7].

In the present work, fruit of the CI-tolerant and sensitive cultivars Natura and Sinatra were stored at chilling (4 °C) and non-chilling (20 °C) temperature for 14 days, and their transcriptomic profiles examined by RNA-Seq. The analysis of the transcriptomic changes in response to cold stress between these cultivars contrasting in their sensitivity to chilling will provide new insight into important mechanisms and processes related to resistance against low temperature stress. Moreover, the outcomes of this study will be the basis for future studies aimed to identify markers involved in cold tolerance, which will surely improve the breeding programs of this crop.

## Methods

### Plant material and postharvest treatments

The commercial zucchini hybrids Natura (Enza Zaden) and Sinatra (Clause-Tezier) were grown under the same greenhouse condition in Almeria, Spain (FEMAGO S.L.). After harvest, fruits of each cultivar were stored in chambers at 4 °C and 20 °C during 14 days. Fruits were divided into three replicates per cultivar and storage period (0 and 14 days), each consisting in 6 fruits of similar size. After storage, weight loss, electrolyte leakage, and chilling injury-index were measured, and the exocarp tissue of each replicate was frozen in liquid nitrogen and stored at − 80 °C.

### Weight loss and chilling-injury index

The percentage of weight loss of each fruit was calculated as: % weight loss = (W_i_ − W_f_)/W_i_ × 100, being W_i_ the initial fruit weight and W_f_ the final fruit weight. Chilling injury index of the fruit surface was evaluated in fruit stored at 4 °C using a subjective scale of visual symptoms previously described [1]: 0 = no pitting, 1 = slight (10% or less), 2 = medium (10–20%), and 3 = severe pitting (> 20%). CI index was determined using the following formula: Ʃ (pitting scale (0–3) × number of corresponding fruit within each class)/total number of fruit estimated.

### Electrolyte leakage

Electrolyte leakage was measured as described [14]. Briefly, exocarp of zucchini fruit was separated with a vegetable peeler and 10 discs were taken from each replicate with an 11 mm diameter stainless steel cork borer. Each replicate was rinsed with 50 mL of deionized water three times for 3 min. After being incubated for 30 min and shaken at 100 rpm in 50 mL of deionized water, this solution was measured for conductivity at room temperature using a conductimeter (Consort C860 provided with a conductivity electrode Consort SK10T, Consort nv, Belgium). Total conductivity was determined after boiling the flasks for 10 min and cooling at room temperature. The electrolyte leakage was expressed as percentage of total conductivity.

### Measurement of lipid peroxidation

Lipid peroxidation was determined as malondialdehyde (MDA) content using the procedure previously described [15], with some modifications. Exocarp ground in liquid nitrogen was homogenized (1:4, *w*/*v*) in 20% (w/v) trichloroacetic acid (TCA) and butylated hydroxytoluene was added to a final concentration of 0.67%. The homogenate was centrifuged at 4 °C and 10,000×g for 15 min. The supernatant was mixed with 0.5% (w/v) thiobarbituric acid (TBA) in 20% TCA in proportion 1:4 (*v*/v). The mixture was heated at 95 °C in a water bath for 30 min, cooled immediately in ice to stop the reaction, and centrifuged at 4 °C and 4000×g for 10 min. Absorbance of supernatant was measured at 532 and 600 nm. MDA content was calculated by subtracting the non-specific absorption at 600 nm from the absorption at 532 nm and using a standard curve. Results were expressed as nmol MDA g^− 1^ of fresh weight.

### Determination of H_2_O_2_ content

H_2_O_2_ content was assayed as described [16]. Zucchini exocarp was ground in liquid nitrogen and homogenized with 0.1% (*w*/*v*) TCA (1:4, w/v). After centrifugation at 4 °C and 12,000×g for 15 min, the supernatant was collected. The reaction mixture consisted of 0.25 mL supernatant, 0.25 mL 100 mM potassium phosphate buffer (pH 7) and 1 mL 1 M KI. The reaction was developed for 1 h in darkness and the absorbance measured at 390 nm. The amount of H_2_O_2_ was calculated using a standard curve and expressed as μmol H_2_O_2_ g^− 1^ of fresh weight.

### RNA extraction

Total RNA was extracted from the exocarp of 6 fruits for each replicate as reported [17], treated with RNAse-Free DNAse and purified using RNasy^®^ MiniElute™ Cleanup columns (Qiagen, Hilden, Germany). The quality and quantity of RNA was determined by agarose gel electrophoresis and NanoDrop Lite spectrophotometer (Thermo Fisher Scientific, MA, USA).

### Sequencing data processing and gene expression analysis

RNA samples were sequenced using Illumina Hiseq2000 at Boyce Thompson Institute (Ithaca, NY, USA). The quality of the single reads generated by Illumina was checked using FastQC (http://www.bioinformatics.babraham.ac.uk/projects/fastqc/). In order to obtain high-quality data, the raw reads were pre-processed and trimmed using the software NGS_CRUMBS (https://bioinf.comav.upv.es/ngs_crumbs/). Through the different utilities the adapters used during the sequencing process were removed, as well as low quality sequences with a Phred quality score Q < 20 and ambiguous sequences with N. Using bowtie2 [18], the high quality reads were mapped against *Cucurbita pepo* transcriptome v3.0 which is included in the genome version 4.1 but was not available when this study was performed [19]. The expression levels were calculated and normalized by the FPKM method with RSEM [20]. Differential expression transcripts were identified using DESeq2 package [21] of the bioconductor package [22, 23]. Transcripts with an adjusted padj (*p*-value adjusted for multiple comparisons using Bejamini-Hochberg method) < 0.05 and a log2 fold change (FC) ± 1.5 based in three biological replicates were considered as DEGs. Principal component and clustering analysis were performed with Mev software [24].

### Gene ontology (GO) terms enrichment analysis

BlastoGO software (v2.8.0) [25] was used for GO term differential analysis using the *Cucurbita pepo* transcriptome v3.0 GO annotation which contains 24,402 annotated unigenes. GO terms enrichment for each data set was calculated by a binomial test model with FDR cut off of 0.05.

### Gene expression analysis by qRT-PCR for RNA-Seq validation

The expression patterns of 10 random DEGs identified by RNA-Seq in this study were validated by quantitative RT-PCR. Primers pairs for each gene (Additional file 1: Table S1) were designed using Primer3 web tool (http://bioinfo.ut.ee/primer3-0.4.0/primer3/). Total RNA was extracted as above. First-strand cDNA was synthesized from 1 μg total RNA using Maxima Reverse Transcriptase (Thermo Fisher Scientific, Rockford, IL, USA). For qRT-PCR, amplifications were run in a 96-well-plates iCycler iQ thermal cycler (Bio-Rad) using iQ SyBr Green Supermix (BioRad). Quantification was performed with the iCycler iQTM associated software (Real Time Detection System Software, version 2.0). The relative gene expression was calculated using non-stored fruit as the calibration sample. *EF-1α* was used as the internal reference gene for normalizing the transcript profiles following the 2^-ΔΔCt^ method [26].

### Statistical analysis

The experimental design was completely randomized. Data were subjected to analysis of variance (ANOVA) or unpaired t-test using Statgraphics Centurion XVI (Statpoint Technologies, Inc., Warrenton, VA, USA). When appropriate, means were separated by Tukey’s HSD test and differences at *p* < 0.05 were considered significant.

## Results

### Fruit physiological parameters and incidence of chilling injury

Natura and Sinatra fruit were stored at 4 and 20 °C during 14 days and postharvest quality parameters including percentage of weight loss, CI index, electrolyte leakage, lipid peroxidation (as MDA content), and H_2_O_2_ were recorded (Table 1). Storage at 4 °C was effective in reducing the fruit weight loss observed in zucchini fruit stored at 20 °C. On the other hand, chilled fruit from Natura and Sinatra showed greater membrane permeability, lipid peroxidation, and H_2_O_2_ content than non-chilled fruit, which presented values similar to fresh harvested fruit in both cultivars. In spite of the clear effect of chilling, the extent of the changes on quality parameters was more pronounced in Sinatra than in Natura. Our data confirmed that cold-stored Sinatra fruit had a greater loss of quality, showing higher weight loss, CI index, electrolyte leakage, lipid peroxidation, and H_2_O_2_ content than Natura fruit.

**Table 1:** Changes on quality parameters in chilling-tolerant (Natura) and chilling-sensitive (Sinatra) zucchini fruit stored at 4 °C and 20 °C during 14 days

	Natura			Sinatra		
	At harvest	20 °C	4 °C	At harvest	20 °C	4 °C
Weight loss (%)	─	9.50 aA	7.13 bB	─	11.13 aA	10.49 aA
CI-Index (0–3)	─	─	0.47 B	─	─	2.18 A
Electrolyte leakage (%)	6.70 bA	6.22 bB	9.42 aB	7.66 bA	8.33 bA	14.28 aA
MDA (nmol gFW^−1^)	42.30 aA	37.84 aA	49.72 aB	42.75 bA	38.60 bA	65.93 aA
H_2_O_2_ (μmol gFW^−1^)	2.29 aA	2.51 aA	2.62 aB	2.08 bB	2.31 bA	3.56 aA

Values are the mean of 3 biological replicates each consisting in 6 fruits. Within each row and cultivar, different lower case letters indicate that means are statistically different (*p* < 0.05) according to Tukey’s HSD test (Electro leakage, MDA, and H_2_O_2_) or un-paired t-test (Weight loss and CI). Within each row and temperature, different capital letters indicate that means are statistically different according to un-paired t-test (*p* = 0.05). *CI* chilling injury, *MDA* malondialdehyde)

### Differential gene expression in the cold stored fruit of the two cultivars

**Fig. 1:**
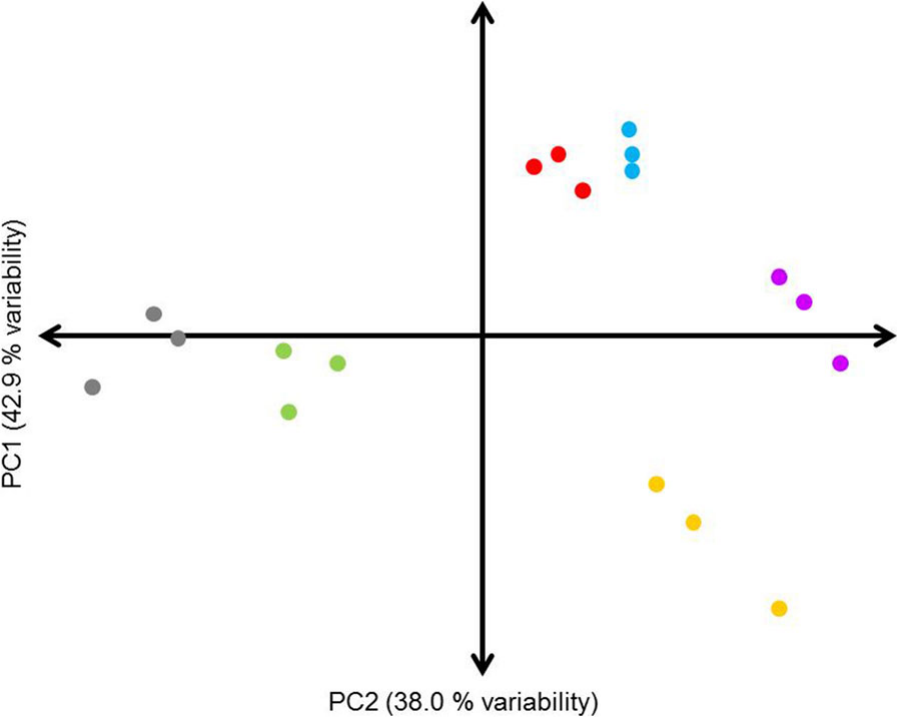
Principal Component Analysis of FPKM normalized gene expression data among three different conditions for Natura and Sinatra. Three independent biological replicates of each condition and cultivar were used. Dark gray, Natura fresh harvested fruit; green, Sinatra fresh harvested fruit; red, Natura fruit stored at 20 °C during 14 days; blue, Natura fruit stored at 4 °C during 14 days; yellow, Sinatra fruit stored at 20 °C during 14 days; purple, Sinatra fruit stored at 4 °C during 14 days

The molecular network regulating zucchini fruit response to chilling was studied by performing a RNA-Seq analysis from exocarp of Natura and Sinatra fruit before and after 14 days of storage at 4 °C and 20 °C. A total of 146 million single reads with an average of 8.1 million reads per sample were generated by Illumina Hiseq2000. After pre-processing and trimming, 133.4 million high quality reads were obtained (an average of 7,412,696 per sample) (Additional file 2: Table S2) and mapped against *Cucurbita pepo* transcriptome v3.0 [19]. To analyze the complexity of the transcriptomic data and to cluster samples according to their gene expression profile, we first performed a principal component analysis (PCA) over the expression data of the 18 biological samples (Fig. 1). The analysis showed that in all conditions the gene expression profile of the three independent biological replicates clustered together; thus the experiment was considered reliable for further analysis. Furthermore, the PCA revealed that at harvest both cultivars presented a different gene expression pattern. Within each cultivar, fresh-harvested fruit clustered away from stored fruit in PC1, which explains 42.9% of the variation. On the other hand, the differences between 4 °C and 20 °C stored fruit in Sinatra are explained by both, PC1 and PC2 (38% of the variation). Interestingly, the changes in the gene expression pattern between Natura chilled and non-chilled fruit were smaller, clustering close together with little separation in either axes.

Gene expression was compared using a pairwise analysis. The comparison of the transcriptome of Natura and Sinatra fruit at harvest (Natura FH/Sinatra FH) revealed 503 DEGs, 262 of which were up-regulated and 241 down-regulated in Natura versus Sinatra (Table 2). The changes in the transcriptomic profiles of the two cultivars were then identified by comparing gene expression values in 4 °C and 20 °C stored fruit (cold-stored/control) in each cultivar independently (Table 2). In Natura, there were 5636 DEGs in cold-stored fruit, from which 2522 were up-regulated (45%) and 3114 down-regulated (55%). Similarly, in cold-stored fruit of Sinatra there were 6623 DEGs, of which 2845 genes were up-regulated (43%) and 3778 genes were down-regulated (57%). To validate the results of transcriptomic profiling, the expression of 10 DEGs was analyzed by quantitative RT-PCR. Linear regression analysis was conducted using Log2 fold change obtained by each approach (Additional file 3: Figure S1). Results showed a significant positive correlation between both methods (R^2^ = 0. 956), thus validating the transcriptomic results obtained by RNA-Seq.

**Table 2:** Transcriptome profiles in Natura and Sinatra fruit exocarp before and after 14 days of storage at 4 °C or 20 °C

		Differentially expressed genes		
Cultivar	Pairwise comparison	Up-regulated	Down-regulated	Total
	Natura FH/Sinatra FH	262	241	503
Natura	CS/Control	2522	3114	5636
	CS/FH	2490	3753	6243
Sinatra	CS/Control	2845	3778	6623
	CS/FH	2634	3530	6164

*FH* fresh harvested fruit, *CS* fruit stored at 4 °C during 14 days, Control, fruit stored at 20 °C during 14 days

**Fig. 2:**
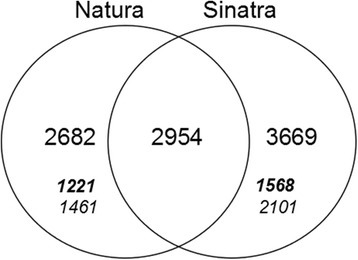
Venn diagram of the differentially expressed genes in fruit from Natura and Sinatra exposed to cold storage (4 °C vs 20 °C). Bold-italic numbers depict the DEGs specifically up-regulated due to cold storage in each cultivar. Italic numbers depict the DEGs specifically down-regulated due to cold storage in each cultivar

DEGs of each cultivar were compared using a Venn diagram to identify specific genes related to cold tolerance or sensitivity (Fig. 2). Results showed that 2682 DEGs were specific to Natura and 3669 were specific to Sinatra. Regarding up-regulated DEGs, 1221 were specific of Natura and 1568 were exclusively identified in Sinatra (45% and 43% respectively). Down-regulated genes differentially regulated in Natura were 1461; while in the case of Sinatra, 2101 were specific, representing 54% and 57% of the DEGs in each cultivar, respectively. The percentage of specific DEGs induced and repressed in response to chilling was similar in both cultivars. On the other hand, Natura and Sinatra shared 2954 DEGs, from which 1264 were induced (43%) and 1640 were down-regulated (55%) in both cultivars. Moreover, there was a group of common DEGs showing opposite cold-specific regulation, i.e. 37 DEGs were up-regulated in Natura and down-regulated in Sinatra, while 13 DEGs were repressed in Natura and induced in Sinatra. The complete list of specific and common cold-regulated genes as well as those found differentially expressed between cultivars at harvest is available in Additional file 4: Table S3.

### Functional analysis of the differential gene expression in fruit of the two cultivars

To explore the biological functions of the cold-induced and repressed DEGs of each cultivar, a gene ontology (GO) enrichment analysis was conducted. Overrepresentation of GO terms was evaluated to correlate different biological processes (BPs), molecular functions (MFs), and cellular components (CCs) with cultivar-dependent chilling response in the exocarp of zucchini fruit.

#### Functional analysis of the differential gene expression in fruit of the two cultivars before storage

**Fig. 3:**
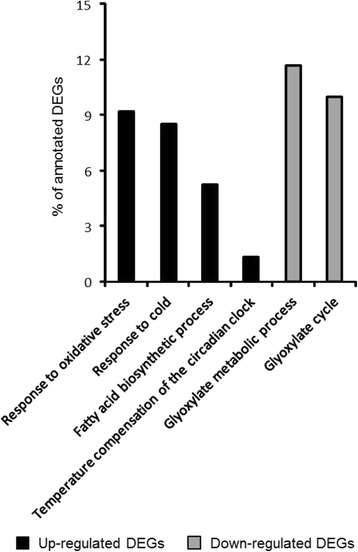
Biological processes (BP) enriched in differentially expressed genes (DEGs) from fresh-harvest Natura fruit compared with fresh-harvest Sinatra fruit

Regarding the comparison between Natura and Sinatra fruit at harvest, 5 GO categories (4 BPs and 1 CC) were up-regulated and 3 (2 BPs and 1 MF) were down-regulated in Natura compared to Sinatra. The BPs represented by the largest number of DEGs up-regulated in Natura fruit compared to Sinatra fruit before storage were ‘response to oxidative stress’, ‘response to cold’ and ‘fatty acid biosynthetic process’, while the three GO categories down-regulated in these fruit were the BPs ‘Glycoxylate metabolic process’ and ‘glycoxylate cycle’ and the MF ‘Isocitrate lyase activity’ (Fig. 3, Additional file 5: Table S4).

#### Functional analysis of the differential gene expression in cold-stored fruit of the two cultivars

**Fig. 4:**
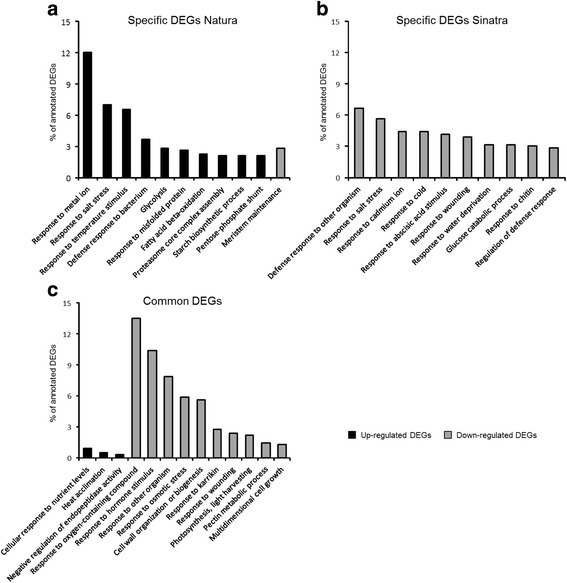
Most enriched biological processes (BP) in percentage of differentially expressed genes (DEGs) specific to Natura (**a**), specific to Sinatra (**b**), or common in both cultivars (**c**) exposed to cold storage (4 °C vs 20 °C)

Figure 4 shows the overrepresented GO terms in DEGs that were specific for the cold tolerant cv. Natura or for the cold-sensitive cv. Sinatra, and in DEGs that were common to both cultivars. In Natura-specific up-regulated DEGs several GO terms were found that belong to abiotic- and biotic-stress responses (16% of up-regulated DEGs) such as response to ‘metal ion’, ‘salt stress’, ‘temperature stimulus’, and ‘defense response to bacterium’ (Fig. 4a). Interestingly, three BPs up-regulated in Natura, i.e. ‘response to misfolded protein’, ‘proteasome core complex assembly’, and ‘proteasomal ubiquitin-dependent protein catabolic processes’, were related to proteolysis (Additional file 6: Table S5). Other BPs overrepresented in Natura were ‘glycolysis’, ‘toxin catabolic process’, and ‘hydrogen peroxide catabolic process’. The functional analysis of up-regulated DEGs in Natura also revealed that the largest MF categories in this cultivar were ‘threonine-type endopeptidase activity’, ‘transferase activity’, and ‘protein domain specific binding’ (Additional file 7: Figure S2A), while ‘cytosol’, ‘chloroplast stroma’, and chloroplast envelope’ were the CCs represented with the largest number of DEGs (Additional file 8: Figure S3A). On the other hand, only two enriched GO terms resulted from the functional analysis of down-regulated DEGs specific of Natura. These GO terms were the BP ‘meristem maintenance’ and the CC ‘cell periphery’ (Fig. 4a and Additional file 8: Figure S3). The former is integrated by DEGs that control the expression of the meristem genes and regulates the response to hormones such as auxins, while the latter includes DEGs whose function are expressed in plasma membrane or cell wall.

The functional analysis of cold response in Sinatra fruit revealed that there were no overrepresented GO terms in up-regulated genes, while there were many terms overrepresented in cold-repressed DEGs (Additional file 9: Table S6). Regarding BPs, 21% of down-regulated DEGs in Sinatra were represented in GO terms related to exposure to biotic and abiotic stress conditions such as ‘defense response to other organism’, ‘response to salt stress’, ‘response to cadmium ion’, ‘response to cold’, ‘response to abscisic acid stimulus’, ‘response to wounding’, and ‘response to water deprivation’ (Fig. 4b). The MFs more overrepresented in down-regulated DEGs were related to ion binding (Additional file 7: Figure S2B) and the largest CCs were the ‘plasma membrane’, ‘plasmodesma’, and ‘chloroplast thylakoid membrane’ (Additional file 8: Figure S3B).

Up-regulated transcripts common to both cultivars were only enriched in three BPs; ‘cellular response to nutrient levels’, ‘heat acclimation’, and ‘negative regulation of endopeptidase activity’ (Fig. 4c). The most overrepresented MFs were related to oxidation-reduction reactions, ‘monooxygenase activity’, and ‘oxidoreductase activity’ (Additional file 7: Figure S2C). On the other hand, the functional analysis of cold-repressed DEGs common to both cultivars (Fig. 4c, Additional file 10: Table S7) revealed that most of the overrepresented BPs were related to response to several stimuli (‘response to hormone stimulus’, ‘response to other organism’, and ‘response to osmotic stress’), cell wall and cuticle biosynthesis (‘pectin metabolic process’, ‘cuticle development’, and ‘wax biosynthetic process’), and photosynthesis and exposition to light (‘photosynthesis, and light harvesting’, ‘non-photochemical quenching’ and ‘anthocyanin accumulation in tissues in response to UV light’). The largest MF in down-regulated DEGs from both cultivars was ‘transferase activity’ (Additional file 7: Figure S2C) and the CCs were ‘plastid envelope’, ‘apoplast’, and ‘plant-type cell wall’ (Additional file 8: Figure S3C).

### Transcription factors

Around 289 and 314 DE transcription factor (TF) genes were identified in Natura and Sinatra fruit, respectively, when 4 °C and 20 °C stored fruit of each cultivar was compared (Additional file 4: Table S3). From these, 47 and 60 were specifically up and down-regulated in Natura respectively; most of them belonged to the zinc finger, NAC, and MYB families. With respect to the cold-sensitive Sinatra, the number of specific down-regulated TFs was much larger (96) than the up-regulated ones (30) and they were part of the zinc finger, WRKY, and AP2/EREBP (including ERFs and CBF/DREBs) families. From these DE TF genes, those belonging to overrepresented BPs identified in our functional analysis are listed in Table 3.

**Table 3:** Expression profile (FPKM) of transcription factors obtained in the functional analysis of differentially expressed genes from Natura and Sinatra fruit before (fresh harvested, FH) and after 14 days of storage at 20 °C and 4 °C

TF family	ID	Description	Natura			Sinatra		
			FH	20 °C	4 °C	FH	20 °C	4 °C
MYB	CUUC76408	Transcription factor MYB3-like	3.66	3.51	16.73	7.48	11.55	78.99
	CUUC60565	Transcription factor MYB21-like	67.90	2.01	79.86	63.86	0.97	88.15
	CUUC61101	MYB-like transcription factor	0.56	1.97	27.34	0.58	0.49	13.62
	CUUC107223	MYB transcription factor	43.98	16.70	4.35	35.69	27.19	7.48
	CUUC92574	MYB transcription factor	27.24	10.09	0.96	62.02	44.99	0.65
	CUUC105130	MYB transcription factor	0.55	9.12	0.28	2.64	49.64	0.63
	CUUC114817	MYB transcription factor MYB6-like	15.93	128.73	2.10	19.16	42.95	3.15
	CUUC114902	Transcription factor MYB21-like	27.37	11.41	0.29	21.23	7.16	0.25
	CUUC99695	Transcription factor MYB44-like	109.74	164.22	28.68	168.20	642.78	21.07
	CUUC100605	Transcription factor MYB44-like	12.21	57.39	5.90	27.76	61.65	4.84
	CUUC96330	Transcription factor MYB44-like	9.40	14.13	0.83	24.65	65.87	0.83
	CUUC100606	Transcription factor MYB44-like	33.63	214.78	17.02	65.88	171.28	21.33
	CUUC100844	Transcription factor MYB86-like	212.66	70.42	15.06	89.09	42.66	8.88
	CUUC61032	MYB-like transcription factor 1	9.91	14.79	2.41	2.90	10.70	2.62
	CUUC88157	MYB-related protein 315-like	42.22	6.72	0.09	23.19	2.69	0.20
	CUUC62256	MYB-related protein 306-like	16.92	20.38	6.74	19.27	49.50	0.82
	CUUC91200	MYB-related protein 306-like	165.85	202.64	48.94	86.80	134.76	40.70
	CUUC97744	MYB-related protein 308-like	17.43	11.96	1.80	8.88	11.43	1.74
	CUUC90701	Transcription repressor MYB6	49.44	78.17	12.15	18.50	78.11	8.59
	CUUC104651	Transcription factor MYB1R1-like	4.07	5.66	6.33	10.71	49.89	2.45
	CUUC107116	Transcription factor MYB1R1-like	271.52	71.70	204.51	281.76	92.69	170.24
	CUUC97743	Transcription factor MYB76-like	0.37	0.16	61.85	0.00	0.04	0.00
MYC	CUUC104046	Transcription factor MYC2-like	19.01	52.12	13.70	27.43	34.69	10.84
	CUUC97393	Transcription factor MYC2-like	0.00	1.65	1.49	0.00	5.43	0.00
	CUUC97395	Transcription factor MYC2-like	0.62	4.07	3.42	1.06	11.91	1.87
	CUUC97396	Transcription factor MYC2-like	1.63	15.37	6.45	3.73	34.58	3.20
AP2/EREBP	CUUC85063	Ethylene-responsive element binding protein (RAP2.3)	6.83	4.75	40.84	11.00	21.25	93.89
	CUUC104934	AP2/ERF domain-containing transcription factor	13.54	8.61	144.59	20.40	171.93	55.08
	CUUC92545	Floral homeotic protein APETALA 2-like	8.78	8.18	1.94	10.96	4.75	2.41
	CUUC60589	Ethylene-responsive transcription factor 1a–like	19.45	74.32	11.80	36.44	232.63	10.58
	CUUC60504	Ethylene-responsive transcription factor 3-like	46.24	156.62	37.86	75.04	564.86	24.47
	CUUC80448	Ethylene-responsive transcription factor 4-like	4.51	21.21	0.84	21.32	318.13	0.38
	CUUC80117	Ethylene-responsive transcription factor ABR1-like	1.72	9.76	0.00	0.78	100.83	0.10
	CUUC86480	Ethylene-responsive transcription factor ERF025-like	1.00	2.79	0.14	1.75	69.38	0.00
	CUUC60600	Ethylene-responsive transcription factor ERF061-like	10.21	6.20	0.12	9.39	40.49	0.11
	CUUC113307	Ethylene-responsive transciptional coactivator-like protein	6.14	41.76	13.51	2.45	12.01	3.73
	CUUC85491	Ethylene-responsive transcription factor 4-like	54.18	10.44	14.05	105.17	57.68	2.81
	CUUC100239	Ethylene-responsive transcription factor 5-like	144.65	269.58	104.81	144.26	264.43	60.98
	CUUC128676	Ethylene-responsive transcription factor ERF025-like	0.00	0.61	0.00	0.00	34.17	0.00
	CUUC67060	Ethylene-responsive transcription factor ERF098-like	2.43	4.96	4.16	3.55	17.70	2.29
	CUUC92773	C-repeat binding factor	14.79	14.92	15.58	17.02	329.14	2.41
WRKY	CUUC78817	WRKY transcription factor	64.33	32.44	0.12	21.16	85.43	0.21
	CUUC78015	WRKY transcription factor 11	9.14	39.95	12.08	8.13	423.96	2.12
	CUUC109151	Probabl WRKY transcription factor 57-like	153.19	176.78	13.09	135.11	110.75	13.70
	CUUC80636	WRKY transcription factor 11	7.99	12.59	12.15	9.14	71.52	3.25
	CUUC101660	WRKY transcription factor 11	91.93	89.85	47.91	70.93	194.23	12.40
	CUUC98190	Probable WRKY transcription factor 15-like	6.36	7.38	3.09	6.81	15.78	1.95
	CUUC95930	Probable WRKY transcription factor 40-like	16.60	50.80	24.11	73.23	756.56	10.15
	CUUC99636	Probable WRKY transcription factor 48-like	24.91	28.80	90.78	17.46	53.54	41.51
Zinc finger	CUUC63321	Zinc finger protein 6-like	4.03	0.07	39.31	2.08	0.24	11.48
	CUUC61049	Zinc finger protein zat10-like	396.63	148.71	1436.86	501.20	1727.79	509.51
	CUUC99174	DOF zinc finger	0.11	11.64	0.27	0.34	80.45	0.21
	CUUC98221	Zinc finger protein	15.27	41.14	13.03	12.66	20.12	5.73
	CUUC96411	Zinc finger protein	11.38	16.64	1.53	19.49	78.29	2.59
	CUUC62446	Zinc finger A20 and AN1 domain-containing stress-associated protein 8-like	795.38	1812.88	84.14	402.13	200.91	59.30
	CUUC114244	Zinc finger protein constans-like 16-like	136.78	22.13	0.91	114.19	47.78	1.13
	CUUC90956	DOF zinc finger	30.54	7.16	7.94	10.04	7.48	1.68
	CUUC99589	Zinc finger A20 and AN1 domain-containing stress-associated protein 5-like	167.88	264.77	204.49	146.95	600.31	101.75
Zinc finger	CUUC94374	Zinc finger protein constans-like 5-like	101.80	54.60	33.48	112.14	122.61	29.87
	CUUC82499	Zinc finger protein ZAT10-like	0.64	6.27	2.85	1.18	12.34	2.68
	CUUC110378	NF-X1-type zinc finger protein NFXL1	8.57	8.57	5.03	8.44	14.71	3.99
	CUUC111731	Zinc finger protein 6-like	0.71	0.00	29.72	0.00	0.00	1.87
	CUUC90133	Zinc finger AN1 domain-containing stress-associated protein 12-like	13.01	23.39	127.98	16.81	70.74	68.77
	CUUC115999	Zinc finger CCCH domain-containing protein 40-like	15.88	15.96	49.68	6.82	16.31	17.54
	CUUC90883	CCCH-type zinc finger protein	157.10	37.39	4.55	86.19	8.55	5.36
	CUUC90884	Zinc finger CCCH domain-containing protein 49-like	465.40	98.87	14.02	240.48	17.12	10.80
	CUUC90885	Zinc finger CCCH domain-containing protein 49-like	661.73	160.02	13.39	308.34	24.90	20.43
	CUUC63652	Zinc finger protein constans-like 9-like	11.31	43.71	8.70	19.90	16.26	8.59
	CUUC107974	Zinc finger protein nutcracker-like	7.45	10.06	2.28	10.43	2.83	1.52
GRAS	CUUC81379	DELLA protein	0.82	0.68	0.28	2.70	6.44	0.07
	CUUC105134	DELLA protein GAIP	11.12	9.99	36.80	13.29	10.46	20.50
HSF	CUUC120379	Heat stress transcription factor b-2a-like	5.38	23.54	6.75	5.67	22.41	6.49
	CUUC114014	Heat stress transcription factor c-1-like	2.22	0.00	4.14	0.87	2.10	1.83
Homeobox-leucine zipper	CUUC100213	Homeobox protein ATH1-like	10.83	28.85	0.91	10.23	14.87	2.55
	CUUC94869	Homeobox-leucine zipper protein ATHB-6-like	6.71	34.04	0.22	4.98	20.26	2.54
	CUUC94870	Homeobox-leucine zipper protein ATHB-6-like	30.21	42.74	4.06	39.53	30.61	4.67
	CUUC103438	Homeobox-leucine zipper protein ATHB-7	4.30	8.57	0.55	2.19	9.27	1.27
	CUUC64965	Homeobox-leucine zipper protein ATHB-13-like	162.15	94.10	7.62	196.41	103.56	24.87
	CUUC114435	Homeobox-leucine zipper protein REVOLUTA-like	7.02	7.87	0.45	10.72	2.76	0.23
NAC	CUUC111022	NAC domain-containing protein 2-like	12.90	10.53	7.26	15.12	37.27	5.11
	CUUC111023	NAC domain-containing protein 2-like	44.99	65.62	39.93	52.37	341.98	35.31
	CUUC127119	NAC domain-containing protein 7-like	0.00	0.32	5.03	0.11	0.50	3.64
Auxin	CUUC79119	Auxin response factor 18-like	3.27	8.95	0.20	2.96	6.95	0.18
ASG	CUUC104842	Transcription factor ASG4-like	68.56	64.21	5.37	50.61	23.46	7.22
TCP	CUUC114074	Transcription factor TCP14-like	15.09	29.40	6.93	31.43	22.70	5.66
bZIP	CUUC109603	bZIP transcription factor 17-like	12.50	24.50	37.19	12.39	69.08	19.55
	CUUC105987	bZIP transcription factor bZIP107	3.07	4.04	0.55	5.08	4.85	0.33
Others	CUUC100506	Transcription initiation factor IIB-2	24.24	33.53	16.27	31.19	44.73	13.84
	CUUC100507	Transcription initiation factor IIB-2	7.88	27.61	12.38	7.70	53.61	6.89
Others	CUUC95110	Probable CRR4-associated factor 1 homolog 11-like	57.66	76.32	89.75	80.36	547.40	32.01
	CUUC90180	Probable CRR4-associated factor 1 homolog 11-like	87.95	242.84	192.73	145.45	1057.13	80.70
	CUUC110049	Transcription factor LHW-like	60.23	115.26	25.12	135.27	81.23	35.87

### Putative candidate genes for cold tolerance in zucchini fruit

In order to identify genes more likely to be related to acquisition of chilling tolerance in zucchini, a careful analysis of all DEGs in our dataset was carried out. The selected genes were those showing a differential expression pattern between Natura and Sinatra and were differentially expressed in cold-stored fruit compared to fruit stored at 20 °C and/or compared to freshly-harvested fruit. The selected candidate genes were grouped according to the following metabolic pathways: *carbohydrate and energy metabolism*, *lipid metabolism*, *peptide transport*, *transcription*, and *signal transduction* (Table 4). Their expression patterns and their possible role in cold-tolerance in zucchini fruit will be discussed below.

**Table 4:** Candidate genes likely to be involved in chilling tolerance of zucchini fruit. Expression profile in fruit before (fresh harvested, FH) and after 14 days of storage at 20 °C and 4 °C is shown in FPKMs

		Natura			Sinatra		
		FH	20 °C	4 °C	FH	20 °C	4 °C
Carbohydrate and Energy Metabolism							
CUUC107944	Malate dehydrogenase	15.18	8.09	24.28	10.44	13.40	8.09
Lipid metabolism							
CUUC60795	Phosphatidylinositol:ceramide inositolphosphotransferase 2-like	10.50	80.72	27.58	17.98	194.82	10.87
Peptide transport							
CUUC107903	Peptide transporter PTR3-A-like	53.95	47.18	279.90	35.85	57.65	91.19
CUUC89270	Peptide transporter PTR2-like	9.65	5.05	32.93	8.81	7.48	16.57
CUUC89268	Peptide transporter PTR2-like	2.46	1.28	10.33	2.75	1.62	4.75
Transcription							
CUUC97743	Transcription factor MYB76-like	0.37	0.16	61.85	0.00	0.04	0.00
CUUC105134	DELLA protein GAIP	11.12	9.99	36.80	13.29	10.46	20.50
CUUC104934	AP2 ERF domain-containing transcription factor	13.54	8.61	144.59	20.40	171.93	55.08
CUUC92773	C-repeat binding factor (CBF)	14.79	14.92	15.58	17.02	329.14	2.41
CUUC61049	Zinc finger protein ZAT10-like	396.63	148.71	1436.86	501.20	1727.79	509.51
Signal Transduction							
CUUC94909	14–3-3 protein	25.70	20.27	70.56	20.35	23.34	18.01
CUUC94908	14–3-3 protein	102.54	73.32	231.82	96.27	115.22	110.64
CUUC94906	14–3-3-like protein	37.59	31.54	155.09	28.02	51.27	48.98
CUUC94907	14–3-3-like protein	136.59	65.48	319.09	101.46	161.40	134.28
CUUC111254	14–3-3-like protein	79.77	87.18	256.54	56.72	71.25	123.10

## Discussion

Zucchini fruit is known to be sensitive to cold storage; although the degree of chilling susceptibility is very dependent on the cultivar [6, 9]. Present fruit quality parameters in Natura and Sinatra fruit after cold storage confirmed previous reports indicating that chilling affects the fruit of the two cultivars, although Natura fruit were more tolerant to chilling and withstood cold better than Sinatra fruit. Furthermore, the chilling tolerance induced by diverse postharvest treatments, including preconditioning at moderate temperature before cold storage [12] or individual shrink wrapping [11], correlates with a better fruit antioxidant status. However, a holistic overview of the molecular bases of the zucchini response to chilling is still lacking.

### Transcriptional bases for the differential response of fruit of the two zucchini cultivars to cold storage

The PCA analysis showed a differential transcriptomic response between cultivars; Sinatra fruit stored at 20 °C were clustered away from fruit stored at 4 °C, whereas chilled and non-chilled fruit from Natura were grouped together. Likewise, the number of DEGs in cold-stored fruit compared to fruit stored at 20 °C was higher in cv. Sinatra than in cv. Natura. This result suggests larger transcriptomic changes in the cold-sensitive Sinatra after cold storage. On the other hand, 2954 DEGs were shared among Sinatra and Natura, reflecting that some of the chilling responses to cold storage are common in fruit of both cultivars. In this sense, the ratio of up- and down-regulated genes was also similar in both cultivars, meaning that the number of repressed genes was slightly higher than the number of induced genes. Transcriptomic studies in other species suggest that this ratio of differential gene expression in response to cold may not be related to acclimation but rather to a species dependent response. Thus, in several species such as *Arabidopsis thaliana* or *Camellia sinensis*, it has been reported that in response to cold the number of up-regulated genes was larger [27, 28], whereas in other species such as *Populus simonii* or *Solanum lycopersicum* this number was either lower or the same [29, 30].

#### Molecular mechanisms related to chilling tolerance in zucchini fruit

The functional analysis of DEGs in zucchini fruit before storage revealed that the response to abiotic stresses (‘response to cold’, ‘response to oxidative stress’ and ‘regulation of the circadian clock by temperature’) was up-regulated in Natura fruit when compared to Sinatra. Interestingly, the most overrepresented BPs in cold-induced genes from Natura fruit after cold storage were also those related to abiotic (‘response to metal ion’, ‘response to salt stresses, and ‘response to temperature stimulus’) and biotic (‘defense response to bacterium’) responses to stress conditions. On the other hand, in the sensitive cultivar Sinatra, BPs related to biotic (‘defense response to other organism’) and abiotic (‘response to salt stress’, ‘response to cadmium ion’, ‘response to cold’, ‘response to abscisic acid stimulus’, ‘response to wounding’, and ‘response to water deprivation’) stresses were also overrepresented but the genes were down-regulated. Our data suggest that this differential regulation may be related to the degree of sensitivity of zucchini fruit cultivars to low temperature and may be fundamental in preventing the negative effect of cold storage in the more chilling-tolerant cultivar. Furthermore, our results support previous reports showing a cross-talk between different stresses, as is the case of cassava apical shoots [31], asparagus bean seedlings [32], and table grapes [33] exposed to chilling.

Other BPs that were highly enriched from the analysis of specifically up-regulated DEGs in Natura were those involved in the maintenance of energy and redox status, including ‘glycolysis’, ‘fatty acid β-oxidation’ and ‘pentose-phosphate shunt’. Among these DEGs were *pyruvate kinase* (CUUC9946, CUUC104231), *phosphoglycerate kinase* (CUUC91403), *glyceraldehyde-3-phosphate dehydrogenase* (CUUC117427), *ribulose-phosphate 3-epimerase* (CUUC104592), *phosphoribulokinase* (CUUC62669), *citrate synthase* (CUUC110803), *malate dehydrogenase* (CUUC107944), and *fructose-1,6-bisphosphatase* (CUUC111898, CUUC110408). Enhanced expression of these genes may result in activation of carbohydrate catabolism and therefore in a rise of the energy supply for Natura fruit, which agrees with previous reports indicating a higher pool of ATP and high energy status on fruit of this cultivar [7]. Similar findings were revealed by a proteomic analysis in leaves of a cold-tolerant maize genotype [34]. Likewise, Cai and coworkers [35] showed that cold-stored grapes treated with salicylic acid had a better postharvest quality and presented a higher accumulation of an important number of proteins belonging to carbohydrate and energy metabolism pathways. Natura may also increase the ATP production by enhancing respiration, since a delta subunit of two mitochondrial ATP synthase genes (*CUUC101699* and *CUCC101701*) was specifically up-regulated in Natura chilled fruit.

The BPs toxin and hydrogen peroxide catabolic processes were also overrepresented in the chilling-tolerant cultivar. These results suggest that concomitant to an increase in the defense response to stress, the trigger of some mechanism of detoxification may allow Natura fruit to avoid or reduce the oxidative stress associated with cold storage. Several DEGs in this group encode detoxification enzymes such as catalase (*CUUC61495*), peroxidase 2 (*CUUC106379*), or NADPH-dependent thioredoxin reductase 3-like (*CUUC65543*), all of them related to ROS scavenging. This differential expression in Natura would explain the lower H_2_O_2_ levels measured in this cultivar; i.e. cold-stored Natura fruit maintained H_2_O_2_ levels similar to fresh or 20 °C stored fruit, but much lower than those observed in the cold-stored fruit from the sensitive cultivar Sinatra. This is also in accordance with previous reports indicating that the chilling tolerance induced by different postharvest treatments correlated with a high catalase activity and gene expression in zucchini fruit [12, 36].

The RNA-Seq data also point to protein degradation as a key mechanism in the acclimation of cold-tolerant fruit to low temperature. Different processes related to proteolysis (‘response to misfolded protein’, ‘proteasome core complex assembly’, and ‘proteasomal ubiquitin-dependent protein catabolic processes’) were specifically induced in cold-tolerant fruit after 14 days at 4 °C. The ubiquitin-dependent protein degradation involves first the ubiquitination of the target protein by the ubiquitin enzymes in a multi-step process (E1, E2, and E3 enzymes), followed by the degradation of the modified protein by the 26S proteasome, a large multi-catalytic endopeptidase complex [37]. It is generally accepted that the ubiquitin-proteasome system (UPS) allows cells to respond rapidly to intracellular signals and changing environmental conditions such as drought, salinity, and cold stress [38]. The UPS function in stress responses is most communly accomplished through targeting and degradation of a negative regulator in response to a stimulus enabling the activation of signaling pathways required for tolerance of the perceived stress [37]. In Natura, many genes encoding for different subunits of the 26S proteasome (*CUUC61601*, *CUUC111088*, *CUUC114894*, etc.) together with two E3 ubiquitin ligases (*CUUC62721* and *CUUC60741*) were up-regulated in cold-stored fruit, reflecting the importance of the UPS in facilitating the response to chilling conditions.

‘Alternative oxidase’ (AOX) is another interesting functional category overrepresented in Natura fruit (*CUUC91321*, *CUUC91320*, *CUUC91319*). The alternative pathway of electron transport limits ROS production in mitochondria and has been described as a mechanism to prevent chilling damage in cold-sensitive species [39]. In other freshly harvested fruit such as sweet pepper and tomato, different treatments that increase chilling tolerance such as methyl jasmonate or salicylate also induced the expression of AOX and correlated with CI resistance [40, 41]. Similarly, it is likely that Natura fruit could ameliorate the adverse effect of chilling by an increase in the transcription of the *AOX* genes.

#### Molecular mechanisms related to chilling sensitivity in zucchini fruit

The chilling sensitive cultivar Sinatra showed the opposite profile; no enriched GO terms were found in the up-regulated DEGs, but 60 different GO categories were identified among cold-repressed DEGs. Many of these GO terms were related to response to biotic, abiotic and endogenous stimuli including ‘response to cold’, ‘response to salt stress’ or ‘response to ABA stimulus’. This enrichment with cold-repressed genes indicates that, contrary to what happens in Natura, chilling-sensitive fruit are unable or have a lower capacity to cope with the adverse environmental conditions imposed. In particular, several of the down-regulated genes belonging to these BPs were related to Ca^2+^ signaling such as calcium-dependent protein kinases (*CUUC118571*, *CUUC99129*), calmodulin like-proteins (*CUUC93980*, *CUUC65863*), or calmodulin-domain protein kinases (*CUUC118569*) (Additional file 6: Table S6), revealing a general repression of Ca^2+^ signaling in cold-sensitive fruit. These results suggest that similarly to other species, under cold stress conditions Ca^2+^ signaling must be crucial for the acquisition of cold-tolerance [42, 43].

It is important to highlight that one of the first symptoms of chilling is related to damage of the plasma membrane, explaining why some measurements such us electrolyte leakage and lipid peroxidation are considered as good biochemical markers of chilling damage in zucchini fruit [9]. It is notable that nearly 18% of specifically cold-repressed DEGs in Sinatra fruit are associated specifically with the plasma membrane. These DEGs are mainly transporters and protein kinases, which may indicate a loss of functionality due to damage in cold-sensitive fruit after 14 days of storage at low temperature.

### Candidate genes associated with cold tolerance in zucchini fruit

In an attempt to identify genes that may be involved in the mechanisms that control cold tolerance in zucchini fruit, a series of genes that showed differential expression among cultivars were selected. As described previously, energy and carbohydrate metabolism must play an essential role in the tolerance of zucchini to low temperature. From all the DEGs found in the BPs related to maintenance of energy and redox status, the expression of a malate dehydrogenase (MDH) (*CUUC107944*) was specifically induced in fruit of the cold-tolerant cultivar. MDH reaction is involved in central metabolism and redox homeostasis between organelle compartments [44]. In transgenic apple plants, the overexpression of a cytosolic malate dehydrogenase improved their cold and salt tolerance [45], so it is possible that the activation of this enzyme would increase the redox response in zucchini fruit.

Another candidate gene to regulate zucchini response to chilling is the one encoding for phosphatidylinositol:ceramide inositolphosphotransferase 2-like (*CUUC60795*), which catalyzes the transfer of the phosphorylinositol group from phosphatidylinositol to phytoceramide, an essential step in sphingolipid biosynthesis. These molecules have been proposed to play important roles in signal transduction, membrane stability, host-pathogen interactions, and stress responses [46]. The expression of this gene was strongly down-regulated in the sensitive cultivar; however, the role of these lipids in the response of zucchini fruit to low temperature is unknown. In Arabidopsis, their accumulation in response to cold is increased in wild-type (WT) plants with respect to the cold-sensitive double mutant *sld1sld2*, although in the slightly more cold-tolerant mutant *atbi*-*1* the levels of sphingolipids were similar to those of WT [47]. Additionally, the virus-induced silencing in tomato plants of a sphingolipid ∆8 desaturase (*SlSLD*) involved in sphingolipid synthesis induced severe chilling damage after a 4 °C treatment.

Previous studies showed that peptide transport is involved in stress tolerance. It was reported in Arabidopsis that *PTR3-like* gene was overexpressed in response to abiotic [48] and biotic stress [49], and the *AtPTR3* knockout mutant showed higher sensitivity to bacterial pathogen infection and salt stress, suggesting that this transporter protects against biotic and abiotic stress [49]. In zucchini, we also found that the *peptide transporter PTR3-A-like* (CUUC107903) and *PTR2-like* (CUUC89270 and CUUC89268) were specifically up-regulated by low temperature in the chilling tolerant cv. Natura.

The signal transduction mechanisms that follow plant stress perception are usually triggered or mediated by TFs. Therefore, TF genes are good candidates for regulating chilling tolerance in zucchini fruit. A large number of TFs have been related to cold stress as well as to other unfavorable conditions in different species [50–52]. Among them, the *MYB76-like* TF gene (CUUC97743) from zucchini was only detectable in fruit of the cold-tolerant cv. Natura, and its expression increased drastically after cold storage. MYB TFs are crucial in regulating the network responses to abiotic and biotic stresses [53]. *CUUC97743* encodes a polypeptide of R2R3 MYB type that shows a high homology with MYB TFs linked to processes such as epidermal cell differentiation and trichome development in cucumber [54, 55].

Other *TF*s specifically up-regulated in the tolerant cultivar Natura were a *DELLA protein GAIP* (CUUC105134) and *AP2/ERF domain-containing transcription factor* (CUUC104934), whereas five *ERF*s were specifically down-regulated in the sensitive cultivar, including a *C-repeat binding factor* (CBF) (CUUC92773). APETALA2 (AP2)/ethylene-responsive-element-binding protein (EREBP) is a large family of TFs unique to plants that have been implicated in plant responses to stresses such as cold and drought [56, 57]. The family includes ERF (ethylene responsive factors), and DREB (dehydration responsive element binding proteins) involved in ethylene-related responses. The best known cold regulatory signaling pathway is that mediated by CBF/DREB1 [42], a small subfamily of DREB transcription factors which activates the expression of cold-responsive (COR) genes [58]. The expression of Arabidopsis *CBF1*, *CBF2*, and *CBF3* is induced shortly after exposure to low temperature, and their overexpression promotes freezing tolerance in Arabidopsis [59] and other species including tomato, rice, and potato [42], Furthermore, CBF/DREB1s appear to induce the accumulation of DELLA (nuclear growth-repressing proteins), which could be responsible for the growth retardation observed in *CBF/DREB1s* overexpressing plants [60]. Interestingly, a DELLA protein GAIP-coding gene (*CUUC105134*) was specifically up-regulated in Natura, while a DELLA protein-coding gene (*CUUC81379*) was specifically down-regulated in Sinatra concomitant with the down-regulation of the *CBF*, CUUC92773, in this cold-sensitive cultivar.

Regarding C_2_H_2_-type zinc finger transcription factors, *ZAT10-like* (CUUC61049) showed a very interesting expression profile during zucchini postharvest. In Natura its expression was specifically up-regulated in response to low temperature, while in Sinatra this gene was down-regulated with respect to fruit stored at 20 °C, reaching similar expression levels to fresh harvested fruit. Zhu and coworkers [61] also described the expression of a *ZAT10-like protein* gene that was induced in mandarin after 60 days of cold storage. Arabidopsis plants overexpressing ZAT10 exhibited growth retardation and enhanced tolerance to drought, salt, osmotic, heat, and oxidative stress, as well as photo-inhibitory light [62–64]. Although Mittler and coworkers [62] observed that knockout and RNAi mutants of *Zat10* were more tolerant to osmotic and salinity stress, it has recently been reported that this gene functions as a positive regulator in osmotic stress tolerance, withZAT10 phosphorylation being required for its function in Arabidopsis [65]. We propose that the transcription factors identified in our study are promising candidate genes for controlling chilling tolerance in zucchini, especially those specifically induced by low temperature in the cold-tolerant cultivar Natura (MYB76-like, CUUC97743; AP2/ERF-like, CUUC104934; and ZAT10-like, CUUC61049).

An important group of genes related to stress sensing and signal transduction also increased their transcription in Natura compared to Sinatra. Among them, five different genes encoding 14–3-3 like proteins showed higher expression in Natura fruit after 14 days of storage at 4 °C (CUUC94909, CUUC94908, CUUC94906, CUUC94907, CUUC111254). It has been reported that phosphorylated like proteins play an important role in abiotic and biotic stress response pathways by interacting and modulating the activity of target proteins [66], or they may interact with components of hormone signaling pathways, such as the ABA signaling pathway [67], that is known to be active under temperature and other stresses. In zucchini, the tolerance to cold storage during fruit postharvest has been associated with an increase in ABA synthesis [3], and higher expression of 14–3-3-like proteins could be related to this behavior.

## Conclusions

In this work, transcriptomic changes that take place in zucchini after cold storage have been compared in two contrasting cultivars for cold tolerance, Natura and Sinatra. The main response of the cold-tolerant cv. Natura was an induction of the mechanisms common to different stress conditions, whereas that of the cold-sensitive cv. Sinatra was a down-regulation of the same mechanisms. This study also highlights the crucial role of some pathways including carbohydrate and energy metabolism, as well as the regulation of transcription and signal transduction in the acquisition of cold tolerance in zucchini during long-term storage. The data suggest the importance of protein trafficking and degradation in the adaptation of the cold tolerant fruit to low temperature. Among the molecular networks related to chilling tolerance that have been detected by functional analysis of RNA-Seq data, different candidates genes have been selected; these genes could be useful as markers for selection of new lines and hybrids in the current breeding programs of zucchini.

## Additional files

Additional file 1: Table S1. Primers pairs used to perform quantitative RT-PCR. (DOC 35 kb)

Additional file 2: Table S2. RNA-Seq data overview. Numbers of raw and clean reads obtained per sample. (DOC 42 kb)

Additional file 3: Figure S1. Validation of RNA-Seq results. Scatter plot shows simple linear regression and the R-squared (R2) between Log2 fold change values obtained by RNA-seq (X) and qPCR (Y) for 10 randomly selected genes. (DOC 38 kb)

Additional file 4: Table S3. DEGs in fruit stored for 14 days at 4ºC compared to fruit stored at 20 ºC which are specific of Natura fruit (SpecificDEGsNatura Sheet). DEGs in fruit stored for 14 days at 4ºC compared to fruit stored at 20 ºC which are specific of Sinatra fruit (SpecificDEGsSinatra Sheet). DEGs in fruit stored for 14 days at 4ºC compared to fruit stored at 20 ºC and that are common to Sinatra and Natura fruit (CommonDEGs Sheet). DEGs in Natura fruit compared to Sinatra fruit before storage (DEGsNATvsSINHarvest Sheet). (XLSX 692 kb)

Additional file 5:  Table S4. Functional annotation (P value < 0.05 and FDR < 0.05) for differentially regulated genes in fresh harvest Natura respect to Sinatra fruit. (XLSX 14 kb)

Additional file 6: Table S5. Functional annotation (P value < 0.05 and FDR < 0.05) for differentially regulated genes specific of Natura fruit stored 14 days at 4ºC. (XLSX 61 kb)

Additional file 7: Figure S2. Most enriched molecular functions (MF) in percentage of differential expressed genes (DEGs) specific from Natura (A), specific from Sinatra (B), or common in both cultivars (C) exposed to cold storage (4 ºC vs 20 ºC). (DOC 159 kb)

Additional file 8: Figure S3. Most enriched cellular components (CC) in percentage of differential expressed genes (DEGs) specific from Natura (A), specific from Sinatra (B), or common in both cultivars (C) exposed to cold storage (4 ºC vs 20 ºC). (DOC 121 kb)

Additional file 9: Table S6. Functional annotation (P value < 0.05 and FDR < 0.05) for differentially regulated genes specific of Sinatra fruit stored 14 days at 4ºC. (XLSX 81 kb)

Additional file 10: Table S7. Functional annotation (P value < 0.05 and FDR < 0.05) for differentially regulated genes common in Natura and Sinatra fruit stored 14 days at 4ºC. (XLSX 62 kb)

## Abbreviations

1-MCP: 1-Methylcyclopropene
ABA: Abscisic acid
BP: Biological processes
CC: Cellular component
CI: Chilling injury
DEG: Differential expressed gene
FC: Log2 fold change
FDR: False discovery rate
FPKM: Fragments per kilobase of transcript per million mapped reads
GO: Gene ontology
H_2_O_2_: Hydrogen peroxide
MDA: Malondialdehyde
MF: Molecular functions
PCA: Principal component analysis
TBA: Thiobarbituric acid
TCA: Trichloroacetic acid
TF: Transcription factor
