# Supplementary material for: Transcriptomic changes in Cucurbita pepo fruit after cold storage: differential response between two cultivars contrasting in chilling sensitivity
Source: BMC Genomics. 2018 Feb 7;19:125. doi: 10.1186/s12864-018-4500-9 (PMC5804050; doi:10.1186/s12864-018-4500-9)
Supplement: Supplementary file 1 — Primers pairs used to perform quantitative RT-PCR. (DOC 35 kb) [file 12864_2018_4500_MOESM1_ESM.doc]

**Table S1**. Primers pairs used to perform quantitative RT-PCR

| ID | Annotation | Primer sequence (5′to 3′) | |
| --- | --- | --- | --- |
|  |  | Forward | Reverse |
| CUUC61555 | Shikimate o-hydroxycinnamoyltransferase-like | CGAGCGAAGAGGTGAGAAAT | CGCACGACTGTAAACCAAATC |
| CUUC110482 | 3-ketoacyl-CoA synthase 6-like | CTCTCACACTCATCCCCATCA | GGTTTGAAGCAGGCGTAGTC |
| CUUC113611 | 3-ketoacyl-CoA synthase 10-like | GCTATTTTCGTATGGGCTGCT | CTTGCTCGTCTTCTTCCTGGT |
| CUUC91675 | Very-long-chain 3-oxoacyl-CoA reductase 1-like | GTCAATTTCCTCCGACCCCC | CTTTATTTGGATGCTGCCGT |
| CUUC113084 | Very-long-chain enoyl-CoA reductase-like | TAGCCCTGTTGGTGACCTTC | TTGAACAGAAACCCCTTTGG |
| CUUC110487 | AGAMOUS-like MADS-box protein AGL8 homolog | GAATGAGACGGTTTGGGTTG | TGGATGTTGTTGGTGCTTGT |
| CUUC94055 | Homeobox-leucine zipper protein ATHB-7-like | CAAAAGTTAGGCGAGCTTGTT | CTTGATGTCCTCCTCCATTCTC |
| CUUC89353 | 3-ketoacyl-CoA thiolase peroxisomal-like | TGGAGAAGGCAATCAACAGG | GCGAGCAGATCATCAGGATAAG |
| CUUC116183 | Abscisic acid receptor PYL4-like | AACCAGACCTGCTCCGCC | GAGGATGTGGTGTTCGTCGTC |
| CUUC61496 | Catalase | ATATGGTTCTTCCCCGTTCC | TCCTCTAGCATGGACACCCC |
