# Supplementary material for: Transcriptomic changes in Cucurbita pepo fruit after cold storage: differential response between two cultivars contrasting in chilling sensitivity
Source: BMC Genomics. 2018 Feb 7;19:125. doi: 10.1186/s12864-018-4500-9 (PMC5804050; doi:10.1186/s12864-018-4500-9)
Supplement: Supplementary file 2 — RNA-Seq data overview. Numbers of raw and clean reads obtained per sample. (DOC 42 kb) [file 12864_2018_4500_MOESM2_ESM.doc]

**Table S2**. RNA-Seq data overview.

Numbers of raw and clean reads obtained per sample.

| Sample | Raw Reads | Clean Reads |
| --- | --- | --- |
| Nat_0_1 | 12,659,158 | 10,803,478 |
| Nat_0_2 | 4,512,165 | 4,134,801 |
| Nat_0_3 | 7,770,970 | 7,190,063 |
| Nat_14-4_1 | 6,314,531 | 5,721,533 |
| Nat_14-4_2 | 7,505,274 | 6,900,270 |
| Nat_14-4_3 | 5,961,478 | 5,354,911 |
| Nat_14-20_1 | 10,294,750 | 9,638,668 |
| Nat_14-20_2 | 11,930,339 | 10,723,547 |
| Nat_14-20_3 | 5,823,455 | 5,332,304 |
| Sin_0_1 | 7,998,457 | 7,396,745 |
| Sin_0_2 | 6,943,359 | 6,029,519 |
| Sin_0_3 | 7,883,680 | 7,294,641 |
| Sin_14-4_1 | 6,366,545 | 5,901,832 |
| Sin_14-4_2 | 12,852,539 | 12,090,972 |
| Sin_14-4_3 | 5,157,285 | 4,786,731 |
| Sin_14-20_1 | 9,040,889 | 8,152,183 |
| Sin_14-20_2 | 5,702,515 | 5,247,997 |
| Sin_14-20_3 | 11,673,639 | 10,728,328 |
| **Total reads** | **146,391,028** | **133,428,523** |
| **Average/sample** | **8,132,835** | **7,412,696** |
