# Supplementary material for: Transcriptomic changes in Cucurbita pepo fruit after cold storage: differential response between two cultivars contrasting in chilling sensitivity
Source: BMC Genomics. 2018 Feb 7;19:125. doi: 10.1186/s12864-018-4500-9 (PMC5804050; doi:10.1186/s12864-018-4500-9)

Figure S1: Validation of RNA-Seq results. Scatter plot shows simple linear regression and the R-squared (R^2^) between Log^2^ fold change values obtained by RNA-seq (X) and qPCR (Y) for 10 randomly selected genes.


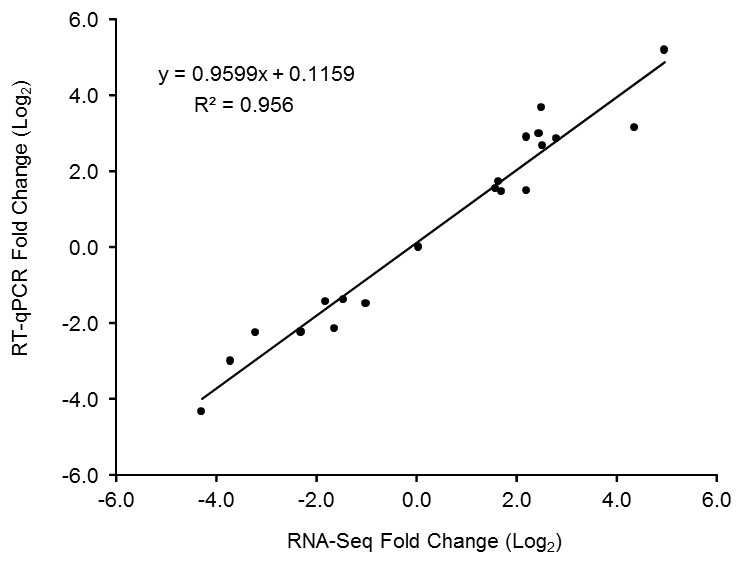

Supplement: Supplementary file 3 — Validation of RNA-Seq results. Scatter plot shows simple linear regression and the R-squared (R2) between Log2 fold change values obtained by RNA-seq (X) and qPCR (Y) for 10 randomly selected genes. (DOC 38 kb) [file 12864_2018_4500_MOESM3_ESM.doc]
