# Supplementary material for: Transcriptomic changes in Cucurbita pepo fruit after cold storage: differential response between two cultivars contrasting in chilling sensitivity
Source: BMC Genomics. 2018 Feb 7;19:125. doi: 10.1186/s12864-018-4500-9 (PMC5804050; doi:10.1186/s12864-018-4500-9)

Figure S2. Most enriched molecular functions (MF) in percentage of differential expressed genes (DEGs) specific from Natura (A), specific from Sinatra (B), or common in both cultivars (C) exposed to cold storage (4 ºC vs 20 ºC).


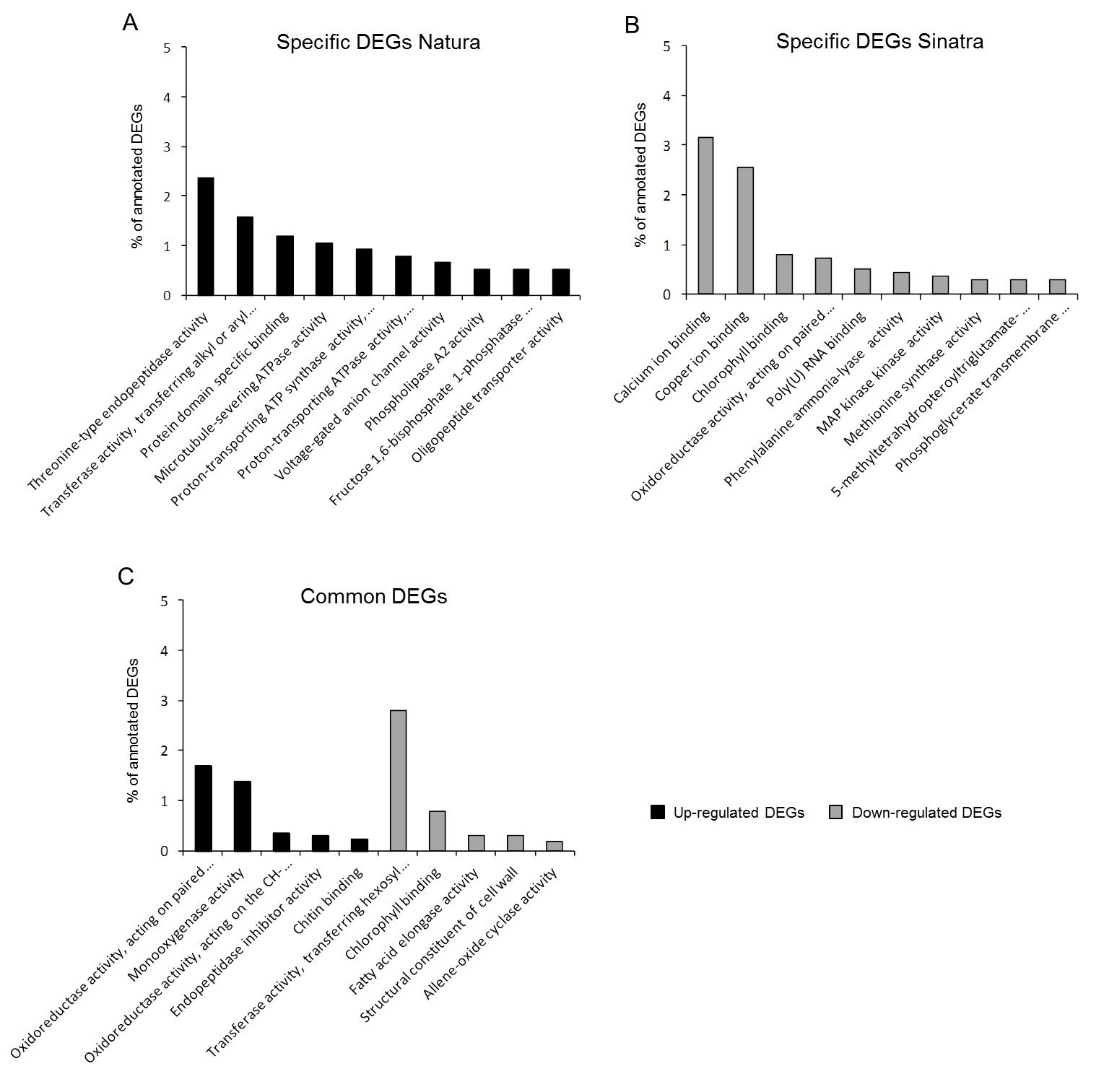

Supplement: Supplementary file 7 — Most enriched molecular functions (MF) in percentage of differential expressed genes (DEGs) specific from Natura (A), specific from Sinatra (B), or common in both cultivars (C) exposed to cold storage (4 ºC vs 20 ºC). (DOC 159 kb) [file 12864_2018_4500_MOESM7_ESM.doc]
