# Supplementary material for: Transcriptomic changes in Cucurbita pepo fruit after cold storage: differential response between two cultivars contrasting in chilling sensitivity
Source: BMC Genomics. 2018 Feb 7;19:125. doi: 10.1186/s12864-018-4500-9 (PMC5804050; doi:10.1186/s12864-018-4500-9)
Supplement: Supplementary file 8 — Most enriched cellular components (CC) in percentage of differential expressed genes (DEGs) specific from Natura (A), specific from Sinatra (B), or common in both cultivars (C) exposed to cold storage (4 ºC vs 20 ºC). (DOC 121 kb) [file 12864_2018_4500_MOESM8_ESM.doc]

Figure S3. Most enriched cellular components (CC) in percentage of differential expressed genes (DEGs) specific from Natura (A), specific from Sinatra (B), or common in both cultivars (C) exposed to cold storage (4 ºC vs 20 ºC).


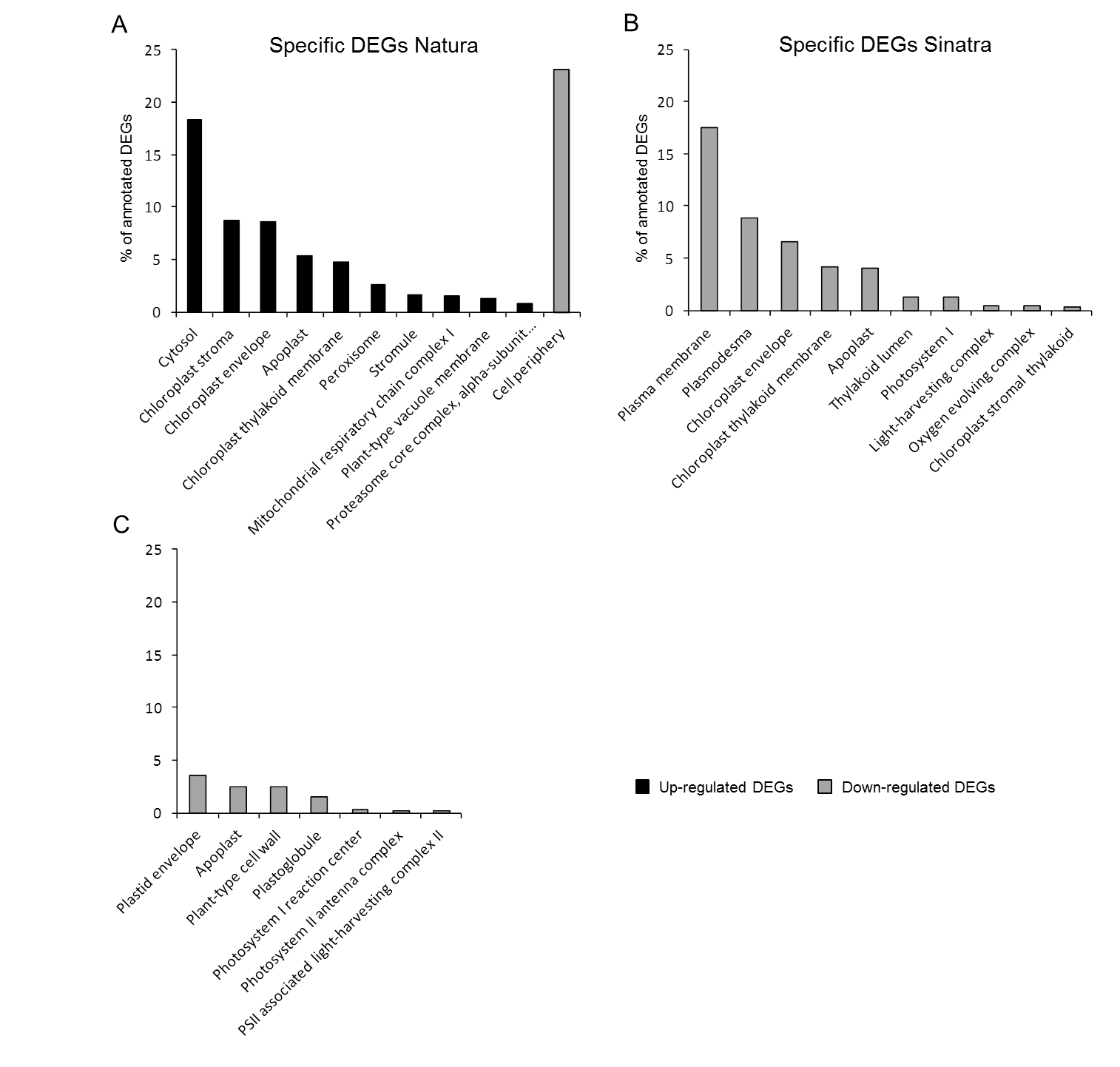


Common DEG
